# Collaboration and health promotion for the health care system – evaluation of the WOL healthcare

**DOI:** 10.1093/eurpub/ckac131.296

**Published:** 2022-10-25

**Authors:** J Schorlemmer, B Jung, C Zeller

**Affiliations:** 1 Institute for Health, FOM Hochschule fuer Oekonomie und Management, Berlin, Germany; 2 Wiesbaden, Germany; 3 Zukunftsherz, Frankfurt, Germany

## Abstract

**Background:**

Health care and social services are industries with special challenges: Constant emotional demands, the shortage of skilled workers is noticeable (in Germany) and special organizational stresses, not only since the Corona pandemic. This study evaluates the Working out Loud (WOL) program for healthcare, which aims to create a learning culture for interdisciplinary collaboration and network-oriented learning and increases growth-oriented thinking at organizational level.

**Methods:**

The sample consists of 51 participants. From 16 persons data could be analyzed in the pre-post-design of the 10-week intervention accompanied by individual coaching. All respondents work in the health care system in Germany. Dependent variables were collected with validated scales for psychological safety, psychological flexibility, cooperative learning, emotional energy, engagement and voice behavior.

**Results:**

Effects of moderate strength were shown for all variables: psychological safety (Mt1= 4.86, Mt2 = 5.45 t(15) =-1.86, p =.083, d = 0.46), psychological flexibility (Mt1= 3.57, Mt2 = 3.82 t(15) = -2.12, p = .051, d = 0.53), cooperative learning (Mt1= 4.63, Mt2 = 4.81 t(15) = -2.18, p =. 045, d = 0.54), emotional energy (Mt1= 2.70, Mt2 = 2.75 t(15) = -0.82, p = .423, d = 0.20), engagement (Mt1= 2.87, Mt2 = 3.05 t(15) = -1.65, p = .119, d = 0.41)and voice behavior (Mt1= 3.84, Mt2 = 4.05 t(15) = -1.64, p = .120, d = 0.41). Correlations are shown for psychological safety with emotional energy (r = .426, p = .012) and job satisfaction (r = .612, p = .000).

**Conclusions:**

The 10-week WOL Healthcare program can strengthen employees in the important area of health promotion and care. The program serves as behavioral prevention and, by empowering individuals, brings about job crafting structural prevention in the workplace. The intervention follows a bottom-up principle, it is an approach for health promotion in the healthcare sector, that can strengthen patient's safety.

**Key messages:**

• Evidence for the effectiveness of a health promotion intervention for health care workers.

• Organizational learning promotes workers health.

